# Small Molecule-directed Immunotherapy against Recurrent Infection by Mycobacterium tuberculosis*[Fn FN2]

**DOI:** 10.1074/jbc.M114.558098

**Published:** 2014-04-07

**Authors:** Debapriya Bhattacharya, Ved Prakash Dwivedi, Mamoudou Maiga, Mariama Maiga, Luc Van Kaer, William R. Bishai, Gobardhan Das

**Affiliations:** From the ‡ Laboratory Medicine and Medical Sciences, College of Health Sciences, University of Kwazulu Natal, Durban 4001, South Africa,; the § Center for Tuberculosis Research, Department of Medicine, The Johns Hopkins University, Baltimore, Maryland 21231-1001, and; the ¶ Department of Pathology, Microbiology and Immunology, Vanderbilt University School of Medicine, Nashville, Tennessee 37232

**Keywords:** Immunology, Immunotherapy, Interleukin, Mycobacterium tuberculosis, T Cell

## Abstract

Tuberculosis remains the biggest infectious threat to humanity with one-third of the population infected and 1.4 million deaths and 8.7 million new cases annually. Current tuberculosis therapy is lengthy and consists of multiple antimicrobials, which causes poor compliance and high treatment dropout, resulting in the development of drug-resistant variants of tuberculosis. Therefore, alternate methods to treat tuberculosis are urgently needed. *Mycobacterium tuberculosis* evades host immune responses by inducing T helper (Th)2 and regulatory T (Treg) cell responses, which diminish protective Th1 responses. Here, we show that animals (Stat-6^−/−^CD4-TGFβRIIDN mice) that are unable to generate both Th2 cells and Tregs are highly resistant to *M. tuberculosis* infection. Furthermore, simultaneous inhibition of these two subsets of Th cells by therapeutic compounds dramatically reduced bacterial burden in different organs. This treatment was associated with the generation of protective Th1 immune responses. As these therapeutic agents are not directed to the harbored organisms, they should avoid the risk of promoting the development of drug-resistant *M. tuberculosis* variants.

## Teaser

**Background:** *M. tuberculosis* evades host-immune-responses by polarizing T helper (Th)2 and regulatory T cell (Treg) responses, which diminish protective Th1 responses.

**Results:** Mice that are unable to generate Th2 cells and Tregs are resistant to *M. tuberculosis* infection. Simultaneous inhibition of these T cell subsets by therapeutic compounds dramatically reduced bacterial burden.

**Conclusion:** Inhibition of Th2 and Treg cells increases Th1 responses that protect against *M. tuberculosis* infection.

**Significance:** As therapeutic agents employed here do not directly act on harbored pathogens, they should avoid generation of drug-resistant *M. tuberculosis* variants.

## Introduction

Tuberculosis represents an enormous burden to global health, with ∼8.7 million new cases and 1.4 million deaths annually (1). *Mycobacterium tuberculosis* is primarily transmitted through the respiratory route and causes active tuberculosis in 5–10% of infected individuals. The host response to the organism is a major determinant of the outcome of infection, and cell-mediated immunity plays a central role in host resistance to this deadly disease. Studies with tuberculosis patients and in animal models have indicated indispensable roles of both CD4^+^ and CD8^+^ T cells in host resistance against *M. tuberculosis* infection (2). Consequently, HIV-infected individuals are profoundly more susceptible to *M. tuberculosis* infection and reactivation of latent infection (3, 4). Collectively, these studies have indicated that CD4^+^ T helper (Th)^2^ cells play a key role in controlling human tuberculosis.

*M. tuberculosis* evades host immune responses by altering the balance of Th cell responses. It is now clear that Th1 cells, which produce IFN-γ and lymphotoxin, play a central role in host resistance to *M. tuberculosis* infection. Therefore, humans or animals with defective Th1 responses are highly susceptible to *M. tuberculosis* infection and disease (5, 6). However, the presence of Th1 cell responses is not sufficient to confer host-resistance against *M. tuberculosis* infection (7, 8). Th2 cells, which produce IL-4, IL-5, and IL-13, assist in disease progression by antagonizing Th1-type immune responses (6). Expression of IL-4 has been detected in human granulomas (9) and is well correlated with the immunopathology predictive of tuberculosis (10, 11). Furthermore, strong Th2 responses have been observed in Bacille Calmette Guérin-vaccinated patients who failed to be protected against *M. tuberculosis* infection (12, 13). Similarly, IL-4-deficient mice display a partial resistance to *M. tuberculosis* infection (14, 15). Therefore, these findings suggest that Th2 cells contribute to susceptibility against *M. tuberculosis* infection. Recent studies have further indicated that *M. tuberculosis* induces antigen-specific, FoxP3-expressing regulatory T cells (Tregs) (16) in the draining lymph nodes (17), which inhibit protective immune responses by hindering IFN-γ production by T cells through production of TGF-β and IL-10 (18–20), thereby promoting disease progression. Therefore, to understand the relative importance of distinct Th cell subsets required to confer resistance against tuberculosis, we have generated animals with impairments in the generation of either Th1 cells, Th2 cells, Tregs, or combinations of these cells. These animals were infected with a low dose (∼110 bacilli/mouse) of the virulent *M. tuberculosis* strain H37Rv through the aerosol route. We demonstrated that animals that are unable to generate Th2 cell responses and Tregs are strongly resistant to *M. tuberculosis* infection. Furthermore, simultaneous inhibition of the differentiation of Th2 cells and Tregs by the therapeutic compounds suplatast tosylate ([3-[[4-(3-ethoxy-2-hydroxypropoxy)phenyl]amino]-3-oxopropyl]dimethylsulfonium 4-methylbenzenesulfonate) and D4476 (4-[4-(2,3-dihydro-1,4-benzodioxin-6-yl)-5-(2-pyridinyl)-1*H*-imidiazol-2-yl]benzene), respectively, substantially reduced bacterial growth in different organs. Therefore, our findings have important implications for the development of improved tuberculosis therapies.

## EXPERIMENTAL PROCEDURES

### 

#### 

##### Ethics Statement

Animal experiments were performed according to the guidelines approved by the Institutional Animal Ethics Committee of International Centre for Genetic Engineering and Biotechnology (ICGEB) (New Delhi, India) and the Department of Biotechnology guidelines (government of India). All mice used for experiments were ethically sacrificed by asphyxiation in carbon dioxide according to institutional and Department of Biotechnology regulations.

##### Bacterial Infections

*M. tuberculosis* H37Rv strain was grown in Middlebrook 7H9 broth (BD Biosciences, Sparks, MD) containing 0.02% Tween 80 to mid-log phase at 37 °C for 3 weeks, aliquoted, and frozen at −80 °C until use. Viable bacterial number was determined on 7H11 agar plates (BD Biosciences) with oleic acid albumin dextrose catalase enrichment (BD Biosciences). Mice were infected via the aerosol route using the nebulizer compartment of an airborne infection apparatus. After 30 min of exposure, the deposition of bacteria was ∼110 bacteria/lung, which was determined by plating the lung homogenates after 24 h of infection. The numbers of viable bacteria in the lung, spleen, and liver of different types of mice were followed at regular time intervals by plating serial dilutions of individual organ homogenates onto nutrient Middlebrook 7H11 agar plates and counting bacterial colony formation after 21 days of incubation at 37 °C. All experiments with *M. tuberculosis* were performed in a biosafety level III facility approved by our Institutional Biosafety Committee of ICGEB (New Delhi, India).

##### Mice

Initially, wild type BALB/c, Stat-6^−/−^, T-bet^−/−^, and CD4-TGFβRIIDN mice were obtained from The Jackson Laboratory, and these strains were subsequently bred in the central animal facility of ICGEB (New Delhi, India). Stat-6^−/−^, T-bet^−/−^, and CD4-TGFβRIIDN mice were intercrossed to obtain Stat-6^−/−^T-bet^−/−^, Stat-6^−/−^CD4-TGFβRIIDN, T-bet^−/−^CD4-TGFβRIIDN, or Stat-6^−/−^T-bet^−/−^CD4-TGFβRIIDN mouse strains. All animals in this study were 6- to 8-week-old females, BALB/c background, and all experiments were carried out as per the approved protocol obtained from the Institutional Animal Ethics Committee.

##### Drug Treatment

Mice were infected with *M. tuberculosis* strain H37Rv as described previously. From day 1 after infection, animals were treated intraperitoneally with D4476 and/or suplatast tosylate (purchased from Tocris Biosciences) at 32 nmol/g up to day 44 after infection.

##### ELISpot Assay

We isolated lung T cells by treatment with collagenase A (1 mg/ml) and DNase concentration (25 μl/ml) at 37 °C for 30 min. The content of the lungs was then crushed through cell strainers with the help of a syringe plunger. Debris was removed by passing through loosely packed nylon wool columns. Cells were washed, and CD4^+^ T cells were enriched with magnetic beads (Miltenyi Biotech). Spleen cells from syngeneic uninfected wild type mice were stimulated with complete soluble antigen (CSA; 50 μg/ml) of H37Rv for 1 h, γ-irradiated (15 Gy), and considered as antigen-presenting cells (APCs). CD4^+^ T cells were cultured in the presence of APCs at a 3:1 ratio for 48 h. Similarly, CD4^+^ T cells obtained from spleen were cultured in the presence of γ-irradiated syngeneic APCs and stimulated with CSA (50 μg/ml) as described above. Antigen-specific IFN-γ-, IL-4-, and IL-17-producing cells in infected lungs and spleen were detected by ELISpot assay (R & D Systems). After 48 h, CD4^+^ T cells co-cultured with γ-irradiated APCs were seeded in antibody-coated plates at an initial density of 5 × 10^4^ cells for lung or 5 × 10^5^ cells for spleen per well. After 18 h, plates were developed, and the frequency and total number of responding cells were determined. Neither cells cultured in the absence of CSA nor cells from uninfected mice produced detectable spots.

##### Cell Staining and Flow Cytometry

CD4^+^ T cells obtained from lung and spleen were cultured in the presence of γ-irradiated syngeneic APCs and stimulated with CSA (50 μg/ml) as described above. The cells (1 × 10^4^) were surface-stained with anti-CD4 (clone GK1.5) PerCP, anti-CD25 (clone PC61.5) APC, and anti-FoxP3 (clone MF23) PE (BD Pharmingen) antibodies. Samples were fixed in a 2% paraformaldehyde solution in PBS and analyzed using a FACSCanto flow cytometer (BD Biosciences) and FlowJo software (Tree Star, Inc.).

For mice treated with suplatast tosylate and D4476 intracellular cytokine staining of splenocytes was performed by activating cells with 50 ng/ml phorbol myristate acetate and 500 ng/ml ionomycin in the presence of 10 μg/ml brefeldin A (Sigma-Aldrich or eBiosciences) added during the last 6 h of culture. Cells were washed twice with PBS and resuspended in a permeabilization buffer (Cytofix/Cytoperm kit; BD Biosciences) and stained with different combinations of the following fluorescently conjugated monoclonal antibodies: anti-mouse CD4 (clone GK1.5) APC (eBioscience), anti-mouse CD4 (clone GK1.5) PE-Cy5 (eBioscience), anti-mouse CD4 (clone GK1.5) PE (eBioscience), anti-mouse CD4 (clone GK1.5) FITC (eBioscience), anti-mouse IFN-γ (clone XMG1.2) APC (Biolegend), anti-mouse IL-4 (clone 11B11) PE (Biolegend), and anti-mouse IL-17A (clone TC11-18H10.1) PE (Biolegend). Fluorescence intensity was measured by flow cytometry (FACSCalibur or FACS CantoII; BD Biosciences), and data were analyzed with FlowJo (TreeStar).

##### Cytokine Assay

Cytokines in the serum and culture supernatant were assayed by a Luminex microbead-based multiplexed assay using commercially available kits according to the manufacturer's instructions (BioPlex, Bio-Rad).

##### Histology

Lungs from mice at 60 days after infection were perfused, removed, and fixed in 4.5% formalin, sectioned, and stained with hematoxylin and eosin.

##### Bacterial Growth Inhibition Assay

*M. tuberculosis* strain H37Rv was grown in 10 ml of Middlebrook 7H9 broth (Becton Dickinson, Sparks, MD) supplemented with 10% ADC (BBLTM, BD Biosciences), 0.05% Tween 80, and 0.2% glycerol at 37 °C under constant shaking. Optical density of the culture was measured at 600 nm using a spectrophotometer (PerkinElmer Life Sciences), and equal numbers of cells from the culture were freshly inoculated in a 30-ml flask containing fresh 7H9 broth such that the initial O.D. of the culture was 0.05. Suplatast tosylate and/or D4476 were added at a dose of 4 nm. Cells were allowed to grow at 37 °C with constant shaking. Optical density of each culture was measured and recorded every 24 h.

##### Statistical Analysis

Data represent mean ± S.D. values of four mice per group per time point for experiments with genetically engineered mice and of six mice per group per time point for experiments with small molecule-mediated immunotherapy. Statistical analyses were conducted using SPSS10 software. Significant differences between the groups were determined by analysis of variance followed by Tukey's multiple comparison tests (SPSS Software). A value of *p* ≤ 0.05 was accepted as an indication of statistical significance. All experiments were repeated twice.

## RESULTS

### 

#### 

##### Development of a Mouse Strain Highly Resistant to M. tuberculosis Infection

**FIGURE 1.**
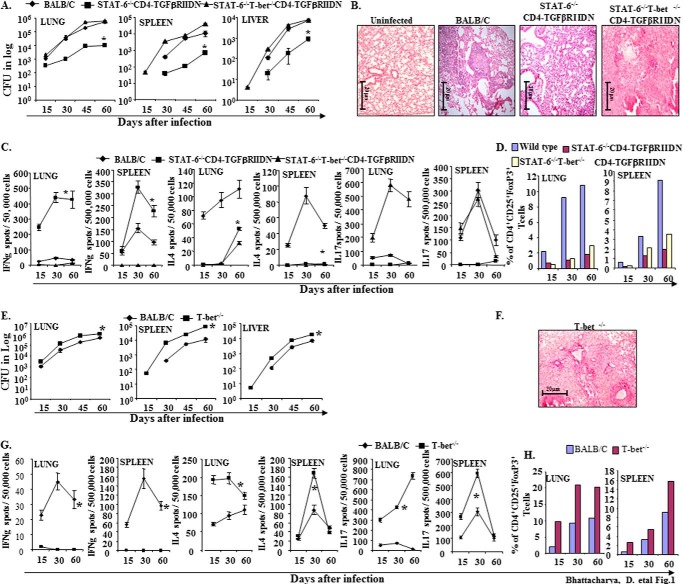
**Stat-6^−/−^CD4-TGFβRIIDN animals are resistant to *M. tuberculosis* infection.** *A*, wild type BALB/c, STAT-6^−/−^CD4-TGFβRIIDN, and STAT-6^−/−^T-bet^−/−^CD4-TGFβRIIDN mice were infected with low dose (∼110 cfu) H37Rv through the aerosol route. Bacterial burdens were determined in the organs and at the time points indicated. Data represent mean ± S.D. values of four mice per group per time point, and the experiment was repeated twice. *B*, histological photomicrographs (×40) of lung sections (6 μm) of wild type BALB/c, STAT-6^−/−^CD4-TGFβRIIDN, and STAT-6^−/−^T-bet^−/−^CD4-TGFβRIIDN mice at 60 days after infection, stained with hematoxylin and eosin. *C*, ELISPOT assay to show the cytokine expression in CD4^+^ T cells in lung and spleen of *M. tuberculosis*-infected wild type BALB/c, STAT-6^−/−^CD4-TGFβRIIDN, and STAT-6^−/−^T-bet^−/−^CD4-TGFβRIIDN mice. *D*, prevalence of CD4^+^CD25^+^FoxP3^+^ T cells in BALB/c, STAT-6^−/−^CD4-TGFβRIIDN, and STAT-6^−/−^T-bet^−/−^CD4-TGFβRIIDN mice was determined by FACS analysis. *E*, wild type BALB/c and T-bet^−/−^ mice were infected with low dose (∼110 cfu) H37Rv through the aerosol route. Bacterial burdens were determined in the organs and at the time points indicated. Data represent mean ± S.D. values of four mice per group per time point, and the experiment was repeated twice. *F*, photomicrographs (×40) of lung sections (6 μm) of T-bet^−/−^ mice at 60 days after infection, stained with hematoxylin and eosin. *G*, cytokine expression in CD4^+^ T cells of lung and spleen from *M. tuberculosis* infected wild type BALB/c and T-bet^−/−^ mice was determined by ELISPOT assay. *H*, prevalence of CD4^+^CD25^+^FoxP3^+^ T cells in pooled T cells obtained from lung and spleen of infected wild type BALB/c and T-bet^−/−^ mice. Significant differences between the groups were determined by analysis of variance followed by Tukey's multiple comparison tests. *Asterisk* indicates statistical significance, *p* ≤ 0.05.

**FIGURE 2.**
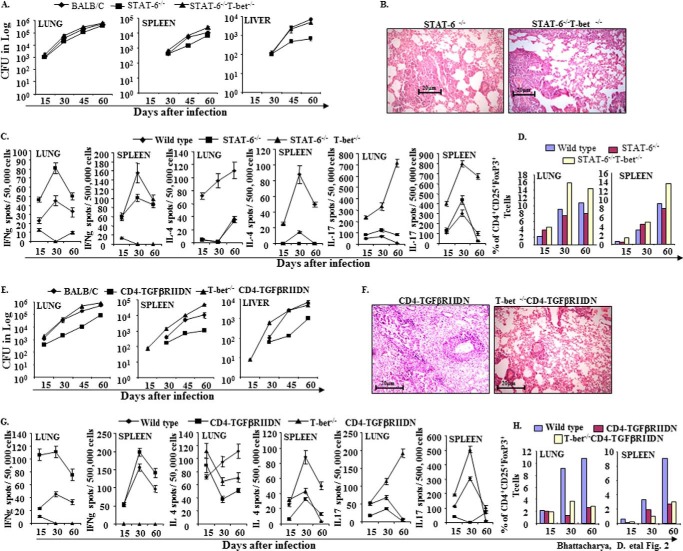
**Stat-6^−/−^ and CD4-TGFβRIIDN animals are partially resistant to *M. tuberculosis* infection.** *A*, wild type BALB/c, STAT-6^−/−^, and STAT-6^−/−^T-bet^−/−^ mice were infected with low dose (∼110 cfu) H37Rv through the aerosol route. Bacterial burdens were determined in the organs and at the time points indicated. Data represent mean ± S.D. values of four mice per group per time point, and the experiment was repeated twice. *B*, histological photomicrographs (×40) of lung sections (6 μm) of STAT-6^−/−^ and STAT-6^−/−^T-bet^−/−^ mice at 60 days after infection. *C*, ELISPOT assay of cytokine expression in CD4^+^ T cells of lung and spleen from *M. tuberculosis*-infected wild type BALB/c, STAT-6^−/−^, and STAT-6^−/−^T-bet^−/−^ mice. *D*, prevalence of CD4^+^CD25^+^FoxP3^+^ T cells was determined by FACS analysis. *E*, wild type BALB/c, CD4-TGFβRIIDN, and T-bet^−/−^CD4-TGFβRIIDN mice were infected with low dose (∼110 cfu) H37Rv through the aerosol route. Bacterial burdens were determined in the organs and at the time points indicated. Data represent mean ± S.D. values of four mice per group per time point, and the experiment was repeated twice. *F*, Histological photomicrographs (×40) of lung sections (6 μm) of CD4-TGFβRIIDN, and T-bet^−/−^CD4-TGFβRIIDN mice at 60 days after infection. *G*, ELISPOT assay of cytokine expression in CD4^+^ T cells of lung and spleen from *M. tuberculosis*-infected wild type BALB/c, CD4-TGFβRIIDN, and T-bet^−/−^CD4-TGFβRIIDN mice. *H*, prevalence of CD4^+^CD25^+^FoxP3^+^ T cells in BALB/c, CD4-TGFβRIIDN, and T-bet^−/−^CD4-TGFβRIIDN mice was determined by FACS analysis.

It has been well established that the Th cell type elicited determines susceptibility or resistance to *M. tuberculosis* infection (5). Thus, to understand the T cell subsets that are required for host resistance or disease progression, we have generated animals that are defective in generating various combinations of Th cell subsets (Table 1). T-bet is a transcription factor required for Th1 cell differentiation (21), whereas Stat-6 is a transcription factor required for Th2 cell differentiation (22), and TGF-β is required for the differentiation of Tregs (23). Therefore, we crossed mouse strains deficient in these transcription factors or with a CD4^+^ T cell lineage-specific TGF-β-signaling deficiency to generate T-bet^−/−^ CD4-TGFβRIIDN, Stat-6^−/−^ CD4-TGFβRIIDN, and T-bet^−/−^Stat-6^−/−^ CD4-TGFβRIIDN mice, which are unable to mount Th1 cells and Tregs, Th2 cells and Tregs, or Th1 cells, Th2 cells, and Tregs, respectively (Table 1). As indicated in Table 1, mice that are deficient in Stat-6 and express a dominant-negative form of the TGF-β receptor in the CD4^+^ T cell lineage (*i.e.* Stat-6^−/−^CD4-TGFβRIIDN mice) are defective in the generation of Th2 cells (22) and Tregs (24, 25). We found that Stat-6^−/−^CD4-TGFβRIIDN mice exhibited high resistance to *M. tuberculosis* infection as determined from the cfu counts in lung, spleen, and liver following infection with virulent *M. tuberculosis* (Fig. 1*A*). We were unable to identify any necrotic granulomas in these animals (Fig. 1*B*). Stat-6^−/−^ and CD4-TGFβRIIDN animals individually showed partial resistance to infection (Fig. 2, *A* and *E*). Interestingly, any of these strains that were also deficient in T-bet, a transcription factor that is critical for Th1 cell differentiation (26), were susceptible to *M. tuberculosis* infection, as shown by bacterial counts (Figs. 1, *A*, and 2, *A* and *E*) and the presence of necrotic granulomas (Figs. 1*B*, and 2, *B* and *F*).

**TABLE 1:** **Genetically engineered mice used in this study and T helper cell subsets produced by them**

Mouse strain	Subsets of T helper cells			
	Th1	Th2	Th17	Treg
WT	+	+	+	+
Stat6^−/−^	+	−	+	+
Tbet^−/−^	−	+	+	+
CD4-TGFβRIIDN	+	+	−	−
Stat6^−/−^Tbet^−/−^	−	−	+	+
Stat6^−/−^ CD4-TGFβRIIDN	+	−	−	−
Tbet^−/−^ CD4-TGFβRIIDN	−	+	−	−
Stat6^−/−^Tbet^−/−^ CD4-TGFβRIIDN	−	−	−	−

##### Dynamics of T Cell Subsets Induced by M. tuberculosis in Distinct Genetically Engineered Mice

To provide insight into the Th cell responses induced by *M. tuberculosis* in distinct genetically engineered animals, we isolated CD4^+^ T cells from the lungs and spleens and determined the frequency of cells with a Th1, Th2, Th17, and Treg cell phenotype. As expected, Stat-6^−/−^CD4-TGFβRIIDN mice induced large numbers of IFN-γ-producing cells, whereas numbers of IL-4-, IL-17-, and FoxP3-positive cells were negligible (Fig. 1, *C* and *D*). Cytokines in serum and in *M. tuberculosis* antigen-stimulated splenocyte cultures similarly revealed that Stat-6^−/−^CD4-TGFβRIIDN mice produced high levels of IFN-γ but were unable to generate IL-4, IL-17, TGF-β, and IL-10 T cell responses (supplemental Table 1). Interestingly, we observed that all groups of animals on the CD4-TGFβRIIDN background exhibited a dramatic increase in IFN-γ-producing cells in both lung and spleen (Fig. 1, *C* and *D*, and Fig. 2, *G* and *H*). Consistent with this finding, we detected large amounts of IFN-γ in the serum and in the supernatant of *M. tuberculosis*-derived CSA-challenged splenocyte cultures from animals that were on a CD4-TGFβRIIDN and/or Stat-6^−/−^ background (supplemental Table 1), whereas mice that were on a T-bet-deficient background showed modestly enhanced numbers of IL-4- and/or FoxP3-positive cells (Fig. 1, *G* and *H*). In sharp contrast, animals that were on a T-bet^−/−^ background exhibited high levels of IL-4-, TGF-β-, IL-10-, and FoxP3-positive cells as well as high levels of IL-4, TGF-β, and IL-10 in serum and in response to CSA stimulation (Fig. 1, *C*, *D*, *G*, and *H*, and Fig. 2, *C*, *D*, *G*, and *H*, and supplemental Table 1). These observations suggested that, in the absence of Th2 cells and Tregs, animals generate sufficient immune responses against *M. tuberculosis* to resist disease development. On the other hand, T-bet^−/−^Stat-6^−/−^CD4-TGFβRIIDN mice are highly susceptible to *M. tuberculosis* infection (Fig. 1*A*), suggesting that the observed resistance of Stat-6^−/−^ CD4-TGFβRIIDN mice to *M. tuberculosis* infection is conferred by potent Th1 responses.

##### Small Molecule-mediated Immunotherapy of M. tuberculosis Infection

**FIGURE 3.**
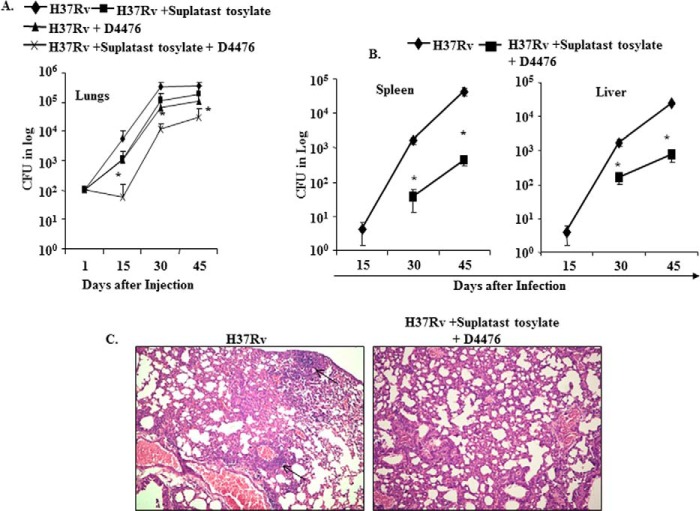
**Simultaneous inhibition of Th2 cytokines and TGF-β protects against *M. tuberculosis* infection.** Wild type BALB/c and mutant mice were infected (110 cfu/mice) through the aerosol route with *M. tuberculosis* (H37Rv). These mice were injected intraperitoneally with D4476 (a TGFβRI inhibitor) and/or suplatast tosylate (a Th2 inhibitor) at 32 nmol/g of body weight starting from the 1st day until the 44th day after aerosol challenge. At different time points (15, 30, 45 days), six mice from each group were sacrificed, and bacterial loads (cfu) were determined in lungs (*A*), spleen, and liver (*B*). *C*, histological photomicrographs (×10) of lung sections (6 μm) at 60 days after infection of the indicated mice, stained with hematoxylin and eosin. *Arrows* indicate granulomatic regions. Significant differences between the groups were determined by analysis of variance followed by Tukey's multiple comparison tests. *Asterisk* indicates statistical significance, *p* ≤ 0.05.

**FIGURE 4.**
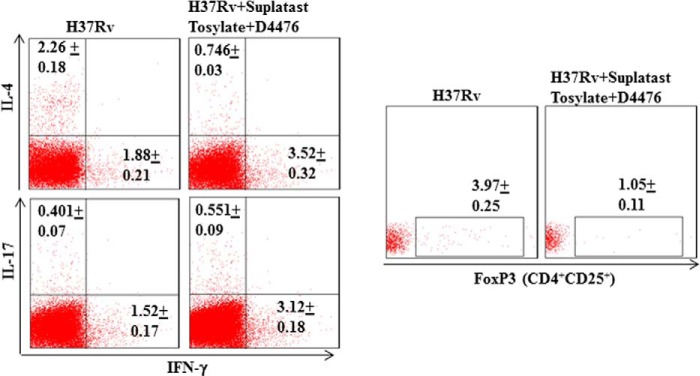
**Intracellular cytokine profiling after combined therapy.** FACS data show the number of IFN-γ-, IL-4-, IL-17, as well as FoxP3-producing cells after combined treatment with suplatast tosylate and D4476.

The preceding sections revealed that simultaneous inhibition of Th2 cells and Tregs might have therapeutic benefits for treatment of tuberculosis. IL-4 and TGF-β play critical roles in the differentiation of Th2 cells and Tregs, respectively. Therefore, we tested whether combined inhibition of IL-4 and TGF-β signaling with small molecules could confer resistance to *M. tuberculosis* infection. Suplatast tosylate disrupts IL-4 signaling and thus inhibits activation and differentiation of Th2 cells (27). Similarly, D4476 inhibits TGF-β signaling (28). We infected wild type BALB/c animals with a low dose of *M. tuberculosis* H37Rv through the aerosol route and then treated the animals with these two drugs. As expected, combined treatment highly diminished bacterial burden in lung, spleen, and liver (Fig. 3, *A* and *B*). Necrotic granulomas were also markedly reduced in animals that received combined therapy, although lungs were modestly inflamed (Fig. 3*C*). Either of these compounds alone was only partially effective. However, combined therapy dramatically enhanced induction of IFN-γ-producing cells and inhibited induction of IL-4-as well as FoxP3-producing cells but had modest effects on IL-17-producing cells (Fig. 4). Interestingly, combined treatment was unable to protect T-bet^−/−^ mice against *M. tuberculosis* infection, suggesting that the therapeutic effects of the small molecules were mediated by promoting protective Th1 cell responses (data not shown).

The studies described in the preceding sections suggested that simultaneous inhibition of IL-4 and TGF-β signaling permits the development of enhanced Th1 responses and facilitates clearance of *M. tuberculosis*. Next, we tested whether these compounds have any direct effects on the growth of the *M. tuberculosis* organisms. As expected, the compounds did not show any significant effect on bacterial growth in liquid culture (supplemental Fig. 1).

TGF-β is an important regulatory cytokine that plays a critical role in the maintenance of immune tolerance (28). TGF-β-deficient animals are embryonically lethal, and CD4-TGFβRIIDN animals display inflammation in multiple organs with enhanced autoactivation of Th1 and Th2 cells (29, 30). Therefore, prolonged inhibition of TGF-β might unmask autoimmunity, which would limit its therapeutic use. However, we did not observe any enhanced inflammation upon infection of CD4-TGFβRIIDN animals with *M. tuberculosis*. Likewise, the short treatment period employed with suplatast tosylate and D4476 did not culminate into signs of excessive inflammation (data not shown).

## DISCUSSION

It is now well accepted that Th cells play a central role in determining susceptibility or resistance to *M. tuberculosis* infection (31). During progression of tuberculosis, *M. tuberculosis* elicits Th2 and Treg cell responses in susceptible hosts, which facilitates disease progression by antagonizing host protective immune responses. Our results suggested that Th1 cells are necessary and sufficient for resistance against tuberculosis, which is in agreement with recently published reports (32). At any given time Th1 and Th2 cells are in a dynamic balance and, thus, mounting Th2 cells decreases host protective Th1 responses. In fact, it has been shown that animals that are unable to mount Th2 responses are partially resistant to *M. tuberculosis* infection (33). Thus, inhibition of Th1 responses by down-regulating IL-12 production in infected cells and eliciting Th2 responses represents a host immune evasion mechanism employed by *M. tuberculosis*. Recently, it has been shown that *M. tuberculosis* facilitates not only Th2 responses but also induces strong Treg cell responses (34). Treg cells have been implicated in inhibiting host protective immune responses (34). TGF-β is required for the differentiation of Treg cells and is produced by the infected cells and by mesenchymal stem cells that infiltrate the site of infection (35, 36). Our data are also consistent with a recent publication (17) providing evidence for profound expansion of Treg cells in the draining lymph nodes during *M. tuberculosis* infection and with previous reports that TGF-β is required for the expansion and maintenance of Tregs. In fact, previous data indicated that mice impaired in TGF-β signaling are partially resistant to *M. tuberculosis* infection (37).

It has been shown that inhibition of Th2 cells or Tregs individually confers partial resistance to *M. tuberculosis* infection (14, 37, 38). Therefore, to test whether combined inhibition of these two subsets has additive therapeutic effects, we generated animals that are defective in both of these subsets (Stat-6^−/−^ CD4-TGFβRIIDN mice). These animals exhibited increased resistance to *M. tuberculosis* infection compared with the individual strains. Therefore, these data suggest that simultaneous inhibition of Th2 cells and Tregs has a superior therapeutic benefit compared with inhibition of the individual subsets alone. Nevertheless, we cannot formally exclude the possibility that deficiency in Stat-6 and TGF-β additionally influences *M. tuberculosis* infection in a manner that is independent of their effects on Th2 and Treg cell responses, respectively.

Inhibiting the differentiation of Th2 cells and Tregs can be achieved by antibodies against IL-4 and TGF-β. However, antibodies are expensive and have several disadvantages such as antigenicity. Therefore, we investigated therapeutic small molecules that could inhibit Th2 cells and Tregs. Suplatast tosylate has been shown to disrupt production of IL-4 and other Th2-type cytokines without impeding IFN-γ production (39). While its molecular mechanism of action has not been fully elucidated, suplatast tosylate inhibits IL-4 production by its direct effects on T cells (40). D4476 is an ALK5 inhibitor and thus prevents Smad3 activation and suppresses TGF-β receptor I gene expression (28). Simultaneous treatment of *M. tuberculosis* infected mice with these two compounds dramatically inhibited bacterial burden in different organs. This mode of therapy was not effective in T-bet-deficient animals, suggesting that it is mainly dependent on the induction of Th1 cell responses. However, it remains possible that these reagents have effects on *M. tuberculosis* infection that are independent of their effects on Th2 and Treg cell responses. Nevertheless, our findings demonstrate that small molecule-directed immunotherapy is effective for the treatment of tuberculosis.

The therapeutic agents employed here are not directed against the harbored pathogen itself. Thus, this mode of therapy should not discriminate between different types of *M. tuberculosis* pathogens, regardless of their antibiotic resistance properties.

Although our therapeutic strategy is novel, we believe that it cannot be employed for a prolonged time period. TGF-β plays an important role in maintaining immune homeostasis and transgenic animals expressing a dominant negative form of TGF-βRII exhibit multi organ inflammation and death (29, 30). Similarly, inhibition of Th2 cells may lead to susceptibility toward parasitic infections (41). Thus, we propose that these compounds could be employed in human patients for relatively short treatment periods. Combining these immunomodulators with antibiotics might reduce the length of treatment and thus avoid the generation of multiple drug-resistant and extremely drug-resistant strains.

In summary, our results have revealed that simultaneous inhibition of Th2 cells and Tregs has great potential for treatment of tuberculosis with small molecules. As these therapeutic agents are not directed toward the harbored pathogens, they should avoid the generation of multiple drug-resistant and extremely drug-resistant variants. In addition, these therapeutic agents are less expensive than conventional recombinant therapeutic agents such as antibodies and should avoid complications associated with recombinant immunotherapeutic agents.

## Supplementary Material

Supplemental Data

## Footnotes

2 The abbreviations used are:

Th: T helper
Treg: regulatory T
CSA: complete soluble antigen
APC: antigen-presenting cell
D4476: 4-[4-(2,3-dihydro-1,4-benzodioxin-6-yl)-5-(2-pyridinyl)-1*H*-imidiazol-2-yl] benzene.
