# Supplementary material for: Small Molecule-directed Immunotherapy against Recurrent Infection by Mycobacterium tuberculosis
Source: J Biol Chem. 2014 Apr 7;289(23):16508–15. doi: 10.1074/jbc.M114.558098 (PMC4047417; doi:10.1074/jbc.M114.558098)
Supplement: Supplemental Data [file supp_289_23_16508__index.html]

Small molecule-directed immunotherapy against recurrent infection by Mycobacterium tuberculosis — Small Molecule-directed Immunotherapy against Recurrent Infection by Mycobacterium tuberculosis — Immunotherapy of Tuberculosis — Supplemental Data 

# Small Molecule-directed Immunotherapy against Recurrent Infection by *Mycobacterium tuberculosis*

## Supplemental Data

**Files in this Data Supplement:**

- Supplemental Table 1 (.doc, 124 KB) - Supplemental Table 1
- Supplemental Figure (.ppt, 166 KB) - Supplemental Figure
- Legends to Supplementary Figure (.doc, 28 KB) - Legends to Supplementary Figure
