# Supplementary material for: Small Molecule-directed Immunotherapy against Recurrent Infection by Mycobacterium tuberculosis
Source: J Biol Chem. 2014 Apr 7;289(23):16508–15. doi: 10.1074/jbc.M114.558098 (PMC4047417; doi:10.1074/jbc.M114.558098)
Supplement: Supplemental Data [file supp_M114.558098_jbc.M114.558098-1.doc]

| **A. IFN-γ** (pg/ml**)** | | | | | | | | |
| --- | --- | --- | --- | --- | --- | --- | --- | --- |
|  | **15 Days** | | **30 Days** | | **45 Days** | | **60 Days** | |
|  | **Serum** | **Antigen specific** | **Serum** | **Antigen specific** | **Serum** | **Antigen specific** | **Serum** | **Antigen specific** |
| Wild type | 16.5±2.1 | 246±22 | 65± 4.32 | 954 ± 382 | 35± 3 | 666 ± 64 | 23± 2.22 | 202 ± 91 |
| STAT-6^-/-^ | 54± 7 | 1110 ± 200 | 69± 8 | 6000 ± 614 | 112± 7 | 2187 ± 542 | 42± 5.3 | 1398 ± 701 |
| T-bet^-/-^ | 0 | 47± 3.1 | 0 | 47± 7 | 0 | 27± 5.5 | 0 | 27± 4 |
| CD4-TGFβRIIDN | 40±5.5 | 711 ± 200 | 73±9 | 5312 ± 714 | 156± 14 | 8111 ± 542 | 271.18±15 | 10567 ± 701 |
| STAT-6^-/-^T-bet^-/-^ | 0 | 12 ± 3 | 0 | 12 ± 7 | 0 | 12 ± 5.55 | 0 | 12 ±4 |
| STAT-6-/-CD4-TGFβRIIDN | 0 | 1420 ± 200 | 0 | 5678 ± 614 | 0 | 9856 ± 542 | 0 | 13987 ± 788 |
| T-bet^-/-^  CD4-TGFβRIIDN | 49±6.7 | 47 ± 15 | 85±15 | 46.9 ±21 | 247.2±35 | 26.9 ± 13 | 348.3±19.3 | 27 ± 7 |
| STAT-6-/-  T-bet-/-  CD4-TGFβRIIDN | 0 | 21 ± 2.3 | 0 | 34 ± 4 | 0 | 26.9 ± 3 | 0 | 28.27 ± 3 |

**Supplemental Information**

**Supplemental Table 1**

| **B. IL-4** (pg/ml**)** | | | | | | | | |
| --- | --- | --- | --- | --- | --- | --- | --- | --- |
|  | **15 Days** | | **30 Days** | | **45 Days** | | **60 Days** | |
|  | **Serum** | **Antigen specific** | **Serum** | **Antigen specific** | **Serum** | **Antigen specific** | **Serum** | **Antigen specific** |
| Wild type | 34.4±3.7 | 205 ± 22 | 56.3±5.1 | 4735 ± 382 | 124.9±13 | 7403 ± 564 | 171±10 | 8403 ± 701 |
| STAT-6^-/-^ | 0 | 16.9± 1.7 | 0 | 23.9 ± 2.8 | 0 | 16 ± 3 | 0 | 3.2 ± 0.9 |
| T-bet^-/-^ | 37.8±3.33 | 1013 ± 345 | 83.6±8.1 | 13456 ± 891 | 165.1±11 | 12620 ± 519 | 276.4±18 | 10967 ± 981 |
| CD4-TGFβRIIDN | 4±0.6 | 177 ± 10 | 14±0.39 | 811 ± 301 | 34±2.1 | 1363 ± 742 | 59±7.23 | 3323 ± 579 |
| STAT-6^-/-^T-bet^-/-^ | 0 | 14.2 ± 2.1 | 0 | 45 ± 4.1 | 0 | 3.2 ± 1.1 | 0 | 3.2 ± 1.1 |
| STAT-6^-/-^CD4-TGFβRIIDN | 24±3.7 | 3.2 ± 0.1 | 67±9 | 3.2 ± 0.9 | 123±23 | 8 ± 0.01 | 191.3±13 | 6.24 ± 0.03 |
| T-bet^-/-^  CD4-TGFβRIIDN | 0 | 487 ± 101 | 0 | 5947 ± 748 | 0 | 7954 ± 1011 | 0 | 8861 ± 589 |
| STAT-6^-/-^  T-bet^-/-^TGFβRIIDN | 0 | 27 ± 4.8 | 0 | 76.12 ± 32.1 | 0 | 117.8± 23 | 0 | 21 ± 2.6 |

| **C. IL-17** (pg/ml**)** | | | | | | | | |
| --- | --- | --- | --- | --- | --- | --- | --- | --- |
|  | **15 Days** | | **30 Days** | | **45 Days** | | **60 Days** | |
|  | **Serum** | **Antigen specific** | **Serum** | **Antigen specific** | **Serum** | **Antigen specific** | **Serum** | **Antigen specific** |
| Wild type | 10±2.8 | 121± 4 | 20.19±1.4 | 821± 243 | 25.2±3.1 | 1209± 701 | 8.26±0.7 | 589 ± 256 |
| STAT-6^-/-^ | 4.3±1 | 1357± 561 | 12±2.7 | 4321± 389 | 24.9±3 | 5759±391 | 3±1.8 | 6185 ± 445 |
| T-bet^-/-^ | 15.5±3 | 355 ± 19 | 19.7±1.7 | 7048 ± 410 | 23±3 | 8488± 379 | 47±3 | 5550 ± 311 |
| TGFβRIIDN | 5±1 | 223 ± 23 | 7.6±2 | 628 ± 89 | 4.±1 | 941 ± 76 | 10±2.1 | 62 ± 7 |
| STAT-6^-/-^T-bet^-/-^ | 5±2 | 209 ± 11 | 15.7±2.1 | 1587 ± 389 | 33.8±2 | 6730± 596 | 43.4±1.7 | 7794 ± 401 |
| STAT-6^-/-^ CD4-TGFβRIIDN | 3.69±0.8 | 67 ± 11 | 8±2 | 335 ± 27 | 17±2.3 | 900 ± 201 | 35±1 | 7 ± 1.7 |
| T-bet^-/-^ CD4-TGFβRIIDN | 7.9±1.1 | 113 ± 11 | 4.9±1.9 | 1759 ± 232 | 3.2±1 | 7444± 199 | 7.1±2 | 4972 ± 343 |
| STAT-6^-/-^  T-bet^-/-^ CD4-TGFβRIIDN | 5±1 | 121± 11 | 13±2.1 | 701 ± 31 | 23±3 | 1700± 567 | 33±1 | 2332 ± 311 |

| **D. IL-10** (pg/ml**)** | | | | | | | | |
| --- | --- | --- | --- | --- | --- | --- | --- | --- |
|  | **15 Days** | | **30 Days** | | **45 Days** | | **60 Days** | |
|  | **Serum** | **Antigen specific** | **Serum** | **Antigen specific** | **Serum** | **Antigen specific** | **Serum** | **Antigen specific** |
| Wild type | 12 ± 1 | 400 ± 89 | 14 ± 0.9 | 700 ± 201 | 15 ± 1.1 | 1121 ± 190 | 21 ± 1 | 700 ± 278 |
| STAT-6^-/-^ | 8 ± 1 | 883 ± 389 | 18 ± 2.01 | 1278 ± 271 | 21 ± 1.16 | 3218 ± 389 | 28 ± 0.87 | 4897 ± 871 |
| T-bet^-/-^ | 10± 0.8 | 306 ± 389 | 18± 1.3 | 2789 ± 378 | 25± 2 | 5597 ± 359 | 30± 99 | 4154 ± 251 |
| CD4-TGFβRIIDN | 3± .01 | 234 ± 65 | 7.5± 1 | 389 ± 187 | 8± 1.2 | 421 ± 67 | 7.2± 1 | 534 ± 189 |
| STAT-6^-/-^T-bet^-/-^ | 12 ± 1 | 140 ± 31 | 25 ± 1.9 | 1769 ± 546 | 24 ± 1 | 4937 ± 231 | 19 ± 0.5 | 5789 ± 235 |
| STAT-6-/- CD4-TGFβRIIDN | 5 ± 0.1 | 201 ± 31 | 7 ± 0.4 | 378 ± 49 | 10 ± 0.9 | 501± 321 | 6 ± 0.3 | 801 ± 275 |
| T-bet^-/-^ CD4-TGFβRIIDN | 2.2 ± 0.1 | 226± 37 | 5.6 ±0.8 | 621 ± 346 | 9.5 ± 0.3 | 3876 ± 311 | 5.4 ± 0.5 | 5234± 435 |
| STAT-6^-/-^  T-bet^-/-^ CD4-TGFβRIIDN | 4 ± 0.1 | 189 ± 31 | 5 ± 0.2 | 1727 ± 546 | 5 ± 0.8 | 3308 ± 111 | 8 ± 0.8 | 4638.7 ± 335 |

| **E. TGF-β** (pg/ml**)** | | | | | | | | |
| --- | --- | --- | --- | --- | --- | --- | --- | --- |
|  | **15 Days** | | **30 Days** | | **45 Days** | | **60 Days** | |
|  | **Serum** | **Antigen specific** | **Serum** | **Antigen specific** | **Serum** | **Antigen specific** | **Serum** | **Antigen specific** |
| Wild type | 45.75±5 | 181 ± 5 | 55.95±3 | 273 ± 11 | 86.15±11 | 330 ± 19 | 101.3±19 | 385 ± 31 |
| STAT-6^-/-^ | 16.5±2 | 262 ± 5 | 45.6±4 | 379 ± 21 | 89.55±13 | 401 ± 29 | 145±13 | 593 ± 53 |
| T-bet^-/-^ | 95±13.09 | 331 ± 19 | 195±21.67 | 497 ± 40 | 290±21 | 704 ± 79 | 305± 31 | 1040 ± 111 |
| CD4-TGFβRIIDN | 8±1.3 | 10 ± 1.21 | 7.7±1.62 | 9 ± 2 | 12±3 | 0 | 11±1.07 | 11 ± 1.76 |
| STAT-6^-/-^T-bet^-/-^ | 75±7 | 248 ± 5 | 140±11 | 280 ± 15 | 195±14.11 | 543 ± 39 | 230±21 | 820 ± 51 |
| STAT-6^-/-^ CD4-TGFβRIIDN | 19±2.36 | 21 ± 2.23 | 14±3.09 | 12 ± 1.73 | 10±.09 | 9 ± 1.11 | 13±2.1 | 8 ± 0.32 |
| T-bet^-/-^ CD4-TGFβRIIDN | 13±1.6 | 12 ± 2.98 | 6.2±0.27 | 8 ± 2.3 | 8.6±2.01 | 14 ± 4.7 | 8±1.31 | 5 ± 0.21 |
| STAT-6^-/-^  T-bet^-/-^ CD4-TGFβRIIDN | 11±0.24 | 5 ± 1.21 | 13±1.9 | 13 ± 2.06 | 8±0.26 | 7 ± 0.87 | 10±0.97 | 11 ± 3.68 |

**Supplementary Table 1. Th1 responses, in the absence of Th2 cells and Treg cells, confer protection against *M. tb* infection.**

For serum cytokines, blood was collected from wild-type BALB/c, Stat-6^-/-^, T-bet^-/-^, Stat-6^-/-^T-bet^-/-^, CD4-TGFβRIIDN, T-bet^-/-^CD4-TGFβRIIDN, Stat-6^-/-^CD4-TGFβRIIDN, and Stat-6^-/-^T-bet^-/-^CD4-TGFβRIIDN mice at 15, 30, 45 and 60 days post infection and serum was prepared and kept at -80°C. For antigen-specific responses, single cell suspensions were obtained from spleen of the indicated mice after 15, 30, 45 and 60 days of infection. RBCs were lysed with Tris/NH_4_Cl, and the cells were washed extensively. Single cell suspensions (5 × 10^6^ cells/well of 24-well plates) were stimulated with CSA of H37Rv (50 μg/ml) for 48 h and supernatants were collected for cytokine assay. Cytokines in the serum and culture supernatant were measured by Luminex microbead-based multiplexed assay. Data are shown as means ± SD of three samples.
