# Supplementary material for: Small Molecule-directed Immunotherapy against Recurrent Infection by Mycobacterium tuberculosis
Source: J Biol Chem. 2014 Apr 7;289(23):16508–15. doi: 10.1074/jbc.M114.558098 (PMC4047417; doi:10.1074/jbc.M114.558098)
Supplement: Supplemental Data [file supp_M114.558098_jbc.M114.558098-2.ppt]

## Slide 1
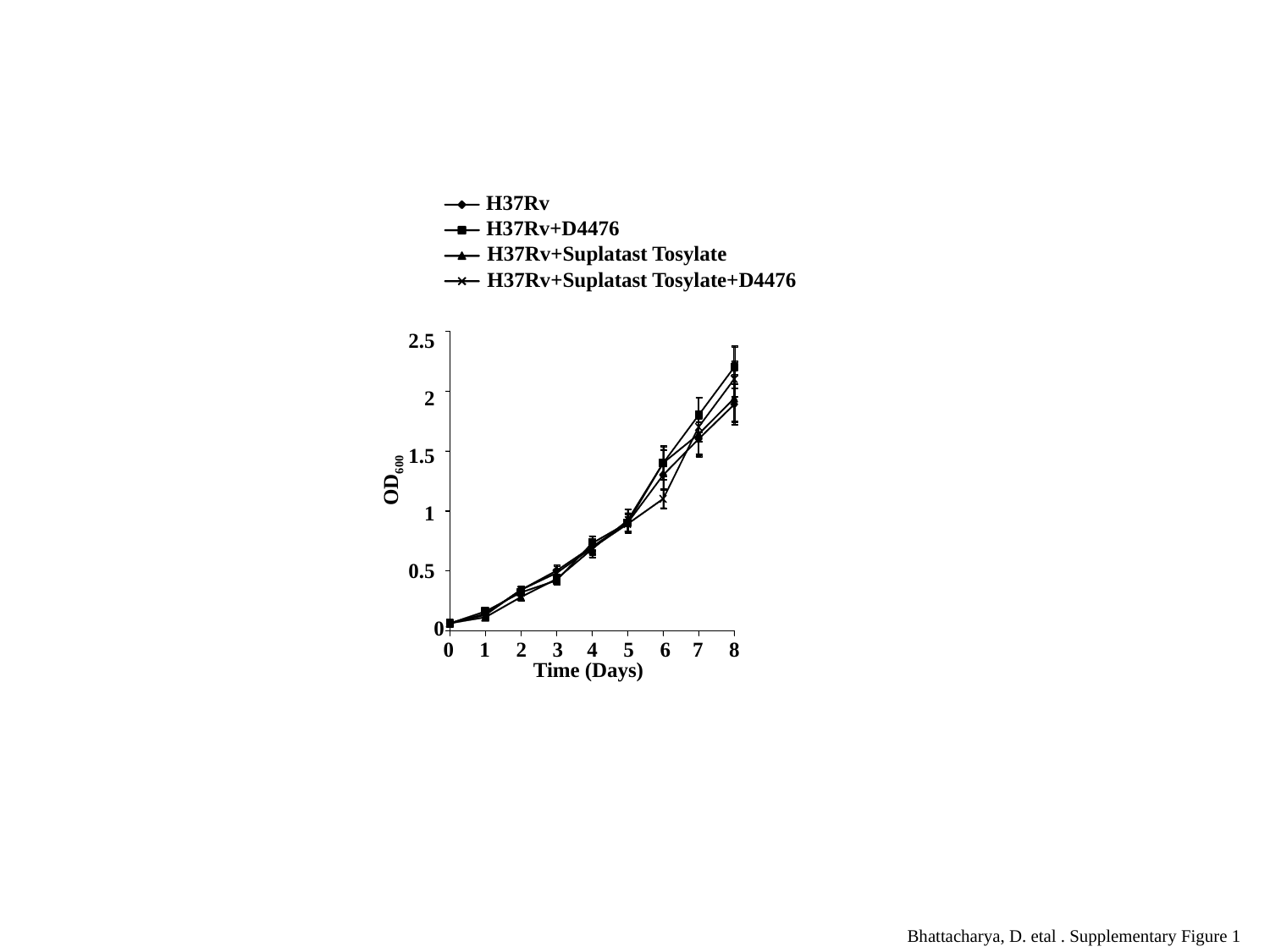

H37Rv
H37Rv+D4476
H37Rv+Suplatast Tosylate
H37Rv+Suplatast Tosylate+D4476
2.5
2
1.5
OD600
1
0.5
0
0
1
2
3
4
5
6
7
8
Time (Days)
Bhattacharya, D. etal . Supplementary Figure 1
