# Supplementary material for: Small Molecule-directed Immunotherapy against Recurrent Infection by Mycobacterium tuberculosis
Source: J Biol Chem. 2014 Apr 7;289(23):16508–15. doi: 10.1074/jbc.M114.558098 (PMC4047417; doi:10.1074/jbc.M114.558098)
Supplement: Supplemental Data [file supp_M114.558098_jbc.M114.558098-3.doc]

***Legends to Supplementary Figures:***

***Supplementary Figure 1. Bacterial growth inhibition assay. OD of the Mycobacterium tuberculosis H37Rv culture at different time points with or without treatment with D4476 and suplatast tosylate.***
